# Study protocol: a dissemination trial of computerized psychological treatment for depression and alcohol/other drug use comorbidity in an Australian clinical service

**DOI:** 10.1186/1471-244X-12-77

**Published:** 2012-07-07

**Authors:** Frances J Kay-Lambkin, Amanda L Baker, Alison Healey, Samantha Wolfe, Aaron Simpson, Michelle Brooks, Jenny Bowman, Steven Childs

**Affiliations:** 1 National Drug and Alcohol Research Centre, University of New South Wales, Sydney, Australia; 2 Centre for Brain and Mental Health Research (CBMHR), University of Newcastle, Newcastle, Australia; 3 School of Psychology, University of Newcastle, Newcastle, Australia; 4 Central Coast Drug and Alcohol Clinical Service, Northern Sydney Central Coast Area Health Service, Sydney, Australia

## Abstract

**Background:**

The rise of the internet and related technologies has significant implications for the treatment of complex health problems, including the combination of depression and alcohol/other drug (AOD) misuse. To date, no research exists to test the real world uptake of internet and computer-delivered treatment programs in clinical practice. This study is important, as it is the first to examine the adoption of the SHADE treatment program, a DVD-based psychological treatment for depression and AOD use comorbidity, by clinicians working in a publicly-funded AOD clinical service. The study protocol that follows describes the methodology of this dissemination trial.

**Methods/design:**

19 clinicians within an AOD service on the Central Coast of New South Wales, Australia, will be recruited to the trial. Consenting clinicians will participate in a baseline focus group discussion designed to explore their experiences and perceived barriers to adopting innovation in their clinical practice. Computer comfort and openness to innovation will also be assessed. Throughout the trial, current, new and wait-list clients will be referred to the research program via the clinical service, which will involve clients completing a baseline and 15-week follow-up clinical assessment with independent research assistants, comprising a range of mental health and AOD measures. Clinicians will also complete session checklists following each clinical session with a client, outlining the extent to which the SHADE computer program was used. Therapeutic alliance will be measured at intake and discharge from both the clinician and client perspectives.

**Discussion:**

This study will provide comprehensive data on the factors associated with the adoption of an innovative, computer-delivered evidence-based treatment program, SHADE, by clinicians working in an AOD service. The results will contribute to the development of a model of dissemination of SHADE, which could be applied to a range of technological innovations.

**Clinical trials registry:**

Australian Clinical Trial Registration Number: ACTRN12611000382976.

## Background

Mounting pressure is exerted on the health system by the increasing prevalence of depression and alcohol/other drug (AOD) misuse. These disorders are ranked 3 and 17 in contribution to the global disease burden, with depression elevated to 1st place and alcohol abuse use to 5^th^ among middle-high income countries such as Australia [1]. Efficacious treatments have been tested with success for both depression [2] and AOD disorders [3], suggesting that this burden can be reduced.

Despite this, the gap between need for and receipt of these treatments is large, particularly for counselling [4], which is often preferred over pharmacotherapy [5]. For example, in the US, 2.1 million people with a 12-month mental disorder did not use services for mental health problems but perceived they had an unmet need [6]. Of these, the highest unmet need was for counselling [6]. Comorbidity, or the co-occurrence of two or more disorders, is the rule rather than the exception in clinical practice [7], with up to 89% of people with AOD use disorders also experiencing depression [8]. The presence of comorbid disorders compounds difficulties in treatment access and provision [9].

Mental health and AOD researchers and clinicians must respond to these issues, by developing and evaluating treatment programs that address depression and AOD use disorders, whilst minimising cost and maximising efficient use of clinician time and client outcomes. Available evidence-based treatments provide for single problems (e.g. depression or alcohol misuse) rather than the comorbidity with which clients typically present [7]. Treatments are often high intensity, require specialist input and training, and are therefore only accessible to a minority of clients [10]. For these reasons, many clinicians are not able, or willing, to implement these interventions in practice.

The increased availability and use of computer/internet-based programs as a supplement to health care is also a potential solution to well-documented treatment accessibility problems [11], particularly among people with depression and AOD use comorbidity. Interactive and multimedia options offer the potential for higher levels of engagement than other self-help modalities [12]. Computers/the internet offer the opportunity for widespread dissemination of treatments, reaching a large audience in a cost effective and timely manner [13]. Experts also suggest that the integration of internet/computer-delivered interventions into practice, will increase adherence to evidence-based treatment protocols, and increase the number of practitioners who can deliver highly specialized psychological treatments [14].

Internet/computerized CBT treatment programs have established efficacy for a range of mental disorders and other health conditions [15]. Our previous work has reported on the efficacy of computerized psychological treatment for concurrent depression and AOD use disorders [SHADE treatment, 16]. In a recent randomized controlled clinical trial involving 97 participants, SHADE computerized treatment was associated with significantly greater reductions in depression relative to a one-session treatment, and equivalent reductions in depression to a face-to-face treatment combining cognitive behavioural therapy (CBT) and motivation enhancement (ME). There was a significant advantage of computerized SHADE for marijuana use over time, with participants in SHADE reporting twice the reduction in marijuana use as the face-to-face condition and approximately five times the reduction as the one-session treatment at 12 month follow-up [16]. Computerized SHADE was also associated with similar reductions in alcohol use over 12 months as the equivalent face-to-face-delivered combination CBT/ME program [16].

Whilst it is generally accepted that internet/computer-delivered CBT programs are efficacious, with some indicating equivalent benefits to face-to-face-delivered programs, there is very little real world research that demonstrates the benefits and acceptability of these programs in practice and service settings [14]. There is some evidence to suggest that, in the US, only 48% of primary care patients would consider using internet-delivered CBT, compared to 91% for traditional face-to-face therapy [14]. However, other evidence suggests that clinicians, including psychologists and CBT practitioners, are more open to using these alternatives as supplements to the care they are able to provide [17]. Consequently, the current study was commenced with the aim of exploring clinician and client uptake, accessibility and response to a computerized CBT/ME treatment for depression and AOD use (SHADE treatment) within a publicly-funded Drug and Alcohol Clinician Service in New South Wales, Australia.

## Methods/design

### Study aims

The purpose of this original research is to test the effectiveness of the SHADE computerized treatment program, from both a clinician and client perspective, within the real world clinical setting. It is hypothesised that clients exposed to the SHADE program will report superior reductions in depression and AOD use relative to those who are not exposed, and that this response may be moderated by primary drug of concern (e.g. marijuana vs. other drug use), coercion into treatment and computer comfort. It is also hypothesised that there will be an association between clinician openness to innovation, clinician computer comfort and the use of SHADE in clinical practice.

### Study setting

This is a real world dissemination trial, conducted within a publicly-funded Drug and Alcohol Clinical Service (DACS) on the Central Coast of New South Wales, Australia. It forms part of the area’s general health service for a population of 306,257. The DACS forms part of a general health service, and provides a range of clinical interventions to residents within the catchment area with AOD use problems. Services include counselling, detoxification (hospital-based and outreach), needle and syringe programs, pharmacotherapy services, a diversion program for people with AOD use problems and legal issues (Magistrates Early Referral Into Treatment, MERIT), and a specialist service targeting clients with a primary drug of concern of marijuana. A central intake service acts as the point of initial contact for access to DACS, with subsequent referrals made to relevant services as appropriate. Client and Clinician participants will be recruited from the counselling services associated with the Central Coast DACS. There are three counselling teams within this service, AOD Counselling, Marijuana Clinic and MERIT. In a study conducted at the NSW Central Coast Cannabis Clinic, it was found that clients on average only attended 2.8 treatment sessions, which is lower than the Drug and Alcohol Counselling Team within the same service which had an average of 4.5 treatment sessions in 2006 [18]. Clients attending the MERIT program are mandated to attend 12 sessions of treatment.

### Participants – clinicians

Clinicians working within the Counselling, Marijuana Clinic and MERIT teams will be invited to participate in the study. At a minimum, these clinicians will have a tertiary education in a counselling-related field, with at least an undergraduate degree in nursing or psychology.

### Participants – clients

All clients, new and ongoing, will be invited to partake in the study. Participants will be aged 18 years and over and residing on the Central Coast and surrounding areas of New South Wales. Participants will consist of individuals attending counselling with primary presenting issues related to substance abuse or dependence. No exclusion criteria will be applied to participants (e.g. comorbid conditions), and it is expected that at least 50% of the clients will be experiencing co-occurring depression or anxiety and many will be poly-drug users. In 2006-7, 2,632 calls were received by the central intake service for the Area Health Region in which the Central Coast DACS is located, with 64% of these being referred to Central Coast DACS. Within the service, 3,329 treatment episodes were commenced, with 73% of clients completing treatment [19]. The majority of these (61%) were for males, aged 20-39 years (51%), with alcohol being the most common primary drug of concern (49%).

### Study design

This study is designed to observe, and not prescribe, the use of the SHADE computerized treatment program within the Central Coast DACS. Ethics approval for the study has been obtained from several relevant Human Research Ethics Committees, led by the Northern Sydney Central Coast Human Research Ethics Committee (08/HARBR/78/79), and including the University of Newcastle Human Research Ethics Committee (H-2008-0271), and the Macquarie University Ethics Review Committee (Human Research, 0806-125M(R)).

#### Clinicians

At information sessions conducted by the authors, clinicians in each team associated with the DACS were introduced to the study and asked to provide consent to participate. Participation involves five activities:

(1) Completion of a baseline focus group discussion regarding the use of innovation in clinical practice.

(2) Completion of a baseline questionnaire regarding their openness to innovation and computer comfort.

(3) Use of the SHADE treatment program with new and ongoing clients in whatever manner they choose, with delivery of the DVD content of the program either contained within the clinic session or provided to clients to complete in their own home in between clinic sessions.

(4) Referral of contact details for new and ongoing clients to the client-data-collection phase throughout the study period, regardless of their exposure to the SHADE treatment program.

(5) Completion of session checklists (see Appendix A) following every counselling session with new and ongoing clients, regardless of their exposure to the SHADE treatment program, and therapeutic alliance measures at intake and discharge for all clients.

Clinicians are provided with a half-day tutorial on the SHADE resource conducted by the research team. The content of this tutorial includes a discussion of the results of the previous clinical trials conducted using the SHADE program, and orientation to the SHADE resource. Clinicians split into groups of two and worked through a different SHADE module on laptops provided by the research team. At the conclusion of this session, clinicians presented each module to the group, and discussed ways in which they felt the module could be integrated into their clinical work at the DACS. Researchers provided their own input into this session where relevant. There was no prescription provided to clinicians as to how, when or with whom the SHADE resource should be used with their clients. However, clinicians suggested that they would use it in the following ways:

(a) “Prescribe” modules for homework in between in-person sessions;

(b) Complete individual modules during the session, sitting side-by-side with clients;

(c) Provide the SHADE resource on DVD-Rom to clients on the wait-list for the DACS;

(d) Use the handouts and worksheets from SHADE to augment discussions about cognitive behavioural skills conducted between clients and clinicians during sessions;

(e) Use of the video and audio material to supplement the relapse prevention group program conducted by the DACS.

### Current/ongoing clients

Following the provision of contact details to the research team via their clinician, current and ongoing clients of the DACS are contacted to discuss consent to participate in the study. Once consent is established, clients complete a baseline and 12-week follow-up assessment delivered over the telephone by research assistants independent from the DACS. Clients are reimbursed $20 AUD for each completed assessment.

### Wait-list clients

New referral to the DACS, via the centralized intake service, who have not been allocated to a clinician, will be contacted by AH, SW or MB (clinicians of the DACS) to discuss study participation and consent to release contact details to the research team. Once these details have been provided to the research team, wait-list clients consent to complete a baseline and 12-week telephone assessment in the same manner as current/ongoing clients. Wait-list clients are reimbursed $20 AUD for each completed assessment.

### The SHADE treatment program

The SHADE treatment program has been described elsewhere [16,20], and incorporates CBT and ME strategies to encourage reductions in depression and AOD use. The program is available in two formats: (i) a 10-session program designed to be completed in a linear fashion, once weekly for 10 weeks, with content pre-programmed for each session; and (ii) a skill module program, where a series of shorter modules are presented based on themes related to depression and AOD use problems (e.g. coping with cravings, taking charge of my thoughts, staying well) arising from the 10-week program. Clients and/or clinicians may choose to focus on just one skill module during a session, without having to complete the other skills and strategies contained in the resource. Both versions of the SHADE program appear on the one DVD-Rom from which the program operates. Text is pitched at a reading age of 14 years, with a voiceover available to read out all text contained in the resource. Video case scenarios guide clients through a range of skills and strategies, and a range of handouts and worksheets are also available for clients/clinicians to print out and use during a session or as a homework activity.

### Assessments

All assessment instruments are widely used in mental health and/or AOD treatment research and practice.

#### Clinicians

Clinicians will participate in a baseline focus group discussion designed to elicit their attitudes and concerns about adopting innovation into their clinical practice in general, and the SHADE treatment program in particular. Table 1 displays the structure of this focus group discussion.

**Table 1:** Clinician focus group protocol

	
(1)	What sources do you use to inform your clinical practice (e.g. journals, workshops, clinical guidelines)?
(2)	What influences you in deciding on when to use a particular strategy, technique, or resource during a session with a client? How do handouts, self-help books and other information for clients fit into this process?
(3)	Have you incorporated any technology into your sessions with clients already? How did you do that, and what was the result?
(4)	Are there any advantages to using technology, e.g. SHADE, as an adjunct to your clinical practice? And what might the disadvantages or concerns be? What are the main issues?
(5)	What are some of the supervision and supports you think that you might need to have in place to assist you in using technology in your clinical practice?

Subsequent to completing the focus group discussion, clinicians complete two further self-report measures:

(1) Innovativeness Scale [21]: a 20-item measure using a 7-point Likert-type scale assessing the likelihood of an individual to adopt innovative strategies in their work.

(2) Computer Opinion Survey [22]: a 26-item measure using a 6-point Likert-type scale, developed as a measure of the trait of computer anxiety rather than the “state” of computer anxiety.

During the course of the study, clinicians complete a session checklist at the conclusion of each session with a client, which outlines the focus and content of the session, including whether or not SHADE or other technologies were used. The checklist was developed by the authors to specifically suit the Central Coast DACS and the range of counselling interventions applied by the clinicians. At the conclusion of each counseling session, clinicians complete a series of tick boxes to report on: the session orientation (initial appointment, assessment, scheduled appointment, non-scheduled client contact, discharge/final session), the intervention applied, whether multimedia was used during the session (if no, why not; if yes, specify the type, e.g. SHADE, and the way in which it was used), whether debriefing was held in relation to the multimedia used with the client, and an approximation of the length of time multimedia was discussed during the session. Please see Appendix A for a copy of the session checklist.

At intake and discharge with a client, clinicians also complete the therapist scale of the Agnew Relationship Measure [23]. This scale asks clinicians to rate, on a 7-point Likert scale, 28 items relating to the extent to which they feel a bond, partnership, confidence, openness, and client initiative are features of the therapeutic relationship with their client.

#### Clients

Following the provision of consent, clients complete the following set of assessment measures at baseline and 12-weeks post-baseline via telephone with a trained research clinician, who is independent of the Central Coast DACS. The following questionnaires take between 30-45 minutes to complete:

(1) Demographics: information includes age, gender, occupational and marital status, children, educational experience, ethnicity and current accommodation arrangements.

(2) Service Utilisation: includes current and previous treatments, including self-reported hospitalisations, attendance at clinics, rehabilitation programmes, contact with community mental health teams, psychologists, psychiatrists, other health professionals, involvement in AOD detoxification and counselling, methadone maintenance, 12-step programmes, use of general practitioners, and use of medication (including compliance).

(3) Opiate Treatment Index [24]: a quantity/frequency index to estimate average daily use of 11 drug types (alcohol, marijuana, heroin, other opiates, amphetamines, cocaine, hallucinogens, barbiturates, tranquilisers, inhalants and tobacco) in the month prior to assessment.

(4) Treatment Motivation Questionnaire: is a 26 item self-report measure, examining four components of motivation including internal and external motivation, help seeking and confidence in treatment. A 7-point Likert scale is used to examine the level of motivation.

(5) Depression Anxiety Stress Scale 21-item version [25]: a 21-item screening tool to for depression, anxiety and stress in the previous 7 days. A 4-point Likert-type scale is used to determine the extent to which a symptom applied to the person.

(6) Global Assessment of Functioning [26]: a clinician-rated assessment of current functioning.

(7) Self-compassion Scale [27]: is a 26-item measure using a 5-point Likert-type scale assessing the extent to which a person expresses self-compassion towards themselves in difficult times.

(8) Agnew Relationship Measure – Client Version [23]: this client-rated measure of therapeutic alliance is similar in content and structure to the therapist-rated version previously described.

We plan to report the cost of delivering the intervention in real world settings and the cost impacts of the outcomes achieved by calibration of selected instruments used in the study (e.g. Quality of Life Scale, Global Assessment of Functioning) with those achieved in other costing studies.

### Sample size calculation

#### Clients

A 50% consent rate is estimated from the 250 eligible clients passing through the Central Coast DACS within the study timeframe (N = 125). Previous research conducted by the authors has achieved consent rates of 50% for participants recruited from the general community [e.g. via media advertisements, 16]. We obtained higher consent rates (i.e. 82%) when previously recruiting directly from DACS [16], however we have estimated our sample size recruitment rates based on the lowest figure. Previous research with the target population has resulted in an 80% retention rate over a 15-week period [16], translating to a final projected sample size of 100 retained participants at the 15-week follow-up for the current study.

#### Clinicians

All clinicians working with the Central Coast DACS are invited to participate in the study, providing a maximum of 19 clinician participants for the trial. Assuming clients are distributed equally between the clinicians, each clinician will see 13 clients during the study period (250/19). Service data from the Central Coast DACS indicate the average occasion of service for clients engaged with the service is three sessions. Assuming a 50% compliance rate with completion of session checklists by clinicians, we estimate having a pool of 342 session checklists for analysis.

### Statistical analyses

#### Clients

For the client sample, primary outcome measures are changes in depression, alcohol and marijuana use between baseline and 12-week follow-up.

Previous research using the SHADE resource among substance users [16] has resulted in effect size differences of 0.42 between clients exposed to the SHADE resource versus not on depression, alcohol and marijuana use. Assuming similar effect size differences will apply to the current study, we estimate that a sample size of 72 is required at 15-week assessment to achieve adequate power (power = 0.81) to detect differences of this order using repeated measures analysis of variance with an alpha level of 0.05 (calculated using G*Power, version 3.1.2). Predictors of alcohol use, marijuana use and depression at 15-weeks relevant to the current study (e.g. client rated therapeutic alliance, internal and external motivation and coerced vs non coerced clients, exposure to SHADE) will be modelled using a linear regression analysis. This sample size will also enable us to examine an effect size of 0.15 for a linear multiple regression for these outcome variables, with up to 6 predictors, an alpha level of 0.05 and a power co-efficient of 0.80 (actual sample size required = 98).

### Clinicians

Given the small sample size of clinicians associated with the DACS, descriptive analyses only will be performed on the clinician measures associated with innovation, computer comfort and reported use of the SHADE resource.

## Discussion

This will be the first real world dissemination trial of the SHADE computerized resource; shown in previous research to be efficacious in reducing depressive symptoms, alcohol and cannabis use among people reporting these comorbid problems, under controlled research trial conditions [16]. We will seek to establish the success with which the SHADE resource is translatable to a publicly-funded Drug and Alcohol Clinical Service. Bennett and Glasgow [28] have recommended the RE-AIM framework [29] as one that includes the critical factors in disseminating technology-based interventions from the research to the clinical setting. The RE-AIM factors are:

(a) Reach: report on the number and percent of participants (eligible, consenting, representativeness);

(b) Effectiveness: the change in key outcomes associated with the intervention;

(c) Adoption: report on the number and representativeness of staff in the clinical setting who consent to participate in the dissemination trial;

(d) Implementation: how the program is delivered, and whether there is consistency in delivery;

(e) Maintenance – individual: long-term effects on individual outcomes; and

(f) Maintenance – setting: sustainability of implementation of the program following completion of the trial.

The current study will cover the first four critical factors (Reach, Effectiveness, Adoption and Implementation), and over the longer term will seek approval to monitor continued use of the SHADE resource in the target Drug and Alcohol Clinical Service. In doing so, it will address several key recommendations important in achieving the goal of widespread dissemination of technology into clinical practice [28].

## Competing interests

None of the authors have any competing interests arising from this research.

## Authors’ contributions

FK-L, AB, AS, AH, SW, MB & SC contributed to the design of the study and developed the protocol. FK-L, AS, AH, SW& MB gained ethical approval for the trial through Northern Sydney Central Coast Human Research Ethics Committee. All authors contributed to manuscript preparation. All authors approved the final manuscript for submission.

## Pre-publication history

The pre-publication history for this paper can be accessed here:

http://www.biomedcentral.com/1471-244X/12/77/prepub
